# A Review of Multidisciplinary Interventions in Atopic Dermatitis

**DOI:** 10.3390/jcm4051156

**Published:** 2015-05-21

**Authors:** Sara C. Spielman, Jennifer S. LeBovidge, Karol G. Timmons, Lynda C. Schneider

**Affiliations:** 1 Division of Immunology, Boston Children’s Hospital, 300 Longwood Ave, Boston, MA 02115, USA; E-Mails: sara.spielman@childrens.harvard.edu (S.C.S.); karol.timmons@childrens.harvard.edu (K.G.T.); lynda.schneider@childrens.harvard.edu (L.C.S.); 2 Harvard Medical School, Boston, MA 02115, USA

**Keywords:** atopic dermatitis, eczema, multidisciplinary, interdisciplinary, education, treatment, quality of life

## Abstract

Multidisciplinary interventions have been developed for patients with atopic dermatitis (AD) and their families, with the aim of improving outcomes such as disease control, adherence, and quality of life. We reviewed the content of different multidisciplinary approaches to intervention for AD and evidence for their impact on key outcome measures. We also provided data from our multidisciplinary outpatient program for pediatric AD. Studies included in the review suggest benefits of multidisciplinary interventions as models of treatment or adjuncts to standard medical care, with a positive impact on outcomes including disease severity and itching/scratching. There were limitations to existing studies, including heterogeneous methods used to assess quality of life outcomes across studies and lack of controlled studies assessing the outcome of clinical care programs. Further research will be useful in assessing the impact of multidisciplinary interventions on important outcomes such as treatment adherence and sleep, identifying the elements of multidisciplinary interventions that are most critical for improved outcomes, and identifying the best candidates for multidisciplinary intervention approaches.

## 1. Introduction

Atopic dermatitis (AD) is one of the most prevalent pediatric skin disorders, affecting 10% to 20% of children and 1% to 3% of adults [1,2]. AD is a chronic condition, characterized by dry, inflamed skin with intense pruritus, or itch. Chronic AD may result in skin lichenification (thickened skin with accentuation of skin markings) [3,4]. The itch-scratch cycle is a hallmark of the condition, with scratching maintaining or exacerbating skin inflammation.

Although there is no cure for AD, it can be treated with a multi-pronged approach including skin hydration (baths, moisturizer use), topical anti-inflammatory medications (corticosteroids and calcineurin inhibitors), wet dressings, antipruritic therapy, anti-bacterial measures (dilute bleach baths, antibiotics), and elimination or control of exacerbating factors, such as irritants, food and environmental allergens, and psychological stress [5]. While such a combination of therapies can help achieve disease control, challenges with consistent adherence are common. Treatment regimens are often time-intensive and complex [6]. Families may experience concerns about side effects of topical anti-inflammatory treatments [7,8,9], as well as difficulty eliciting child cooperation with skincare and maintaining lifestyle modifications such as dietary restrictions due to food allergy [10,11].

The impact of AD on quality of life is well documented, with patients and families reporting the itch-scratch cycle and associated sleep impairments to be the most difficult aspects of the condition [10,11,12,13]. Sleep disruption due to pruritus is common, including difficulty with sleep onset, frequent awakenings, and reduced sleep efficiency [14,15,16,17]. Sleep loss and discomfort due to pruritius may mediate a higher risk for mood, behavior, and attention problems among children with AD [18,19,20]. Additionally, children may experience self-consciousness about physical appearance, as well as stress associated with the chronic nature of the condition [9,21,22]. Psychological stress, in turn, has been shown to be a trigger for AD, leading to a challenging cycle [23,24,25,26].

Given the range of skills required to successfully manage and cope with AD, multidisciplinary interventions have been developed to transfer necessary knowledge and tools to patients and families, with the aim of improving outcomes such as disease control, adherence, and quality of life. We review the content and evidence for different approaches to multidisciplinary interventions in atopic dermatitis, including our outpatient program for children with severe AD and emphasize the common elements of successful programs.

## 2. Methods

Searches using MEDLINE, Pubmed, and Psychinfo were performed using key words including “atopic dermatitis”, “eczema”, “multidisciplinary”, “interdisciplinary”, “treatment”, and “education”. The search was limited to English language studies. We only included studies that tested or described a multidisciplinary intervention for patients with AD or family members of patients with AD. All interventions were required to include clinicians from at least two disciplines (e.g., dermatologist and psychologist). Studies for patients with pediatric AD and adult AD were both included. Given the limited number of reports identified, we included clinical trials, pre-post comparison studies, and descriptive studies. If more than one evaluation of the same intervention by the same group of authors was identified, we included the study with the most rigorous methodology (e.g., randomized controlled trial *vs*. pre-post comparison without control group). References within the identified studies were reviewed for additional studies meeting our inclusion criteria. We categorized interventions according to the treatment modality and patient population. Interventions were categorized as: educational group training programs for pediatric AD; group programs for adult AD; day treatment program for pediatric AD; and outpatient program for pediatric AD.

## 3. Results

### 3.1. Educational Group Training Programs for Pediatric AD

Several studies have been conducted evaluating group-based training programs for parents of children with AD as adjuncts to standard medical care. Staab and colleagues conducted a randomized controlled trial of the Berlin Parental Education Program for parents of children with moderate to severe AD [27]. The training program consisted of 6 weekly 2-h sessions, facilitated by pediatricians and/or dermatologists, psychologists, and dieticians, with opportunities for goal setting, modeling, and group discussion about barriers to treatment. Structured education topics included medical information about AD (definition, symptoms, course, pathophysiology), skincare, stage-related treatment of symptoms, recognition and avoidance of triggers, stress management/relaxation, coping with itching and scratching, management of sleep disturbances, support for child coping (positive self-image, gradual transfer of increased responsibility for skincare to the child), general nutritional guidance, and information about the role of food allergies/diet in AD [27,28]. Parents of children with AD ages 5 to 12 years were randomized into the training program (*n* = 93) or a waitlist control group (*n* = 111). Both groups showed improvement in physician-rated disease severity between the initial evaluation and a 1-year follow-up, with no significant difference between the groups. At follow-up, however, only the treatment group continued with regular use of skincare products. Compared with the control group, participants in the treatment group demonstrated greater increases in the appropriate use of topical steroids and antiseptics based on the severity of the skin, as well as a larger decrease in economic burden due to AD, as measured by the cost of medical visits and prescriptions. Mothers in the treatment group showed a greater increase in satisfaction with the medical treatment of AD and a greater decrease in negative/ruminative thoughts to cope with AD, although there were no other differences between the groups in self-report quality of life or coping measures.

In a large multi-center randomized controlled trial, Staab and colleagues evaluated an adaptation of the Berlin model that stratified participants by child age (German Atopic Dermatitis Intervention Study; GADIS) [29]. A total of 992 families of children with moderate to severe AD were randomized into the training program or a waitlist control group. Parents of children ages 3 months to 7 years attended educational sessions without the child, while children ages 8 to 12 years and their parents attended parallel sessions in separate rooms, and adolescents aged 13–18 years attended sessions independently of their parents (with some sessions allowing for optional parent participation). At a 1-year follow-up, children across ages in the group training condition showed greater improvement in both objective and subjective measures of AD severity than the control group. Compared with the control group, children ages 8–12 in the training group demonstrated greater improvement in coping with and catastrophizing about itch (negative thoughts that are out of control), while adolescents demonstrated greater improvement in catastrophizing about itch. Compared with the control group, parents of children 7 and under in the training group showed improvement in all areas of quality of life (psychosomatic well-being, effects on social life, confidence in medical treatment, emotional coping, and acceptance of the disease), and parents in the 8–12 group in emotional coping, confidence in the medical team, and acceptance of the disease.

Ricci and colleagues evaluated a group program for parents of young children with moderate to severe AD that included selected elements of the Berlin Model [30]. Parents of 30 children ages 5 months to 5 years attended six weekly 90-min sessions led by a pediatric allergist, dermatologist and psychologist; there was no study control group. Topics included general education about AD, skincare, coping with itching and scratching, behavioral management of sleep problems, infant massage (as a form of relaxation during skincare), and food allergy. There were opportunities for parents to ask questions and express concerns about daily management of the child’s disease. Following the conclusion of the program, the authors found improvements in parental self-report of anxiety and depression symptoms, as well as family and child dermatitis-specific quality of life. The majority of parents reported a more “tranquil” attitude towards the disease and an improvement in their relationship with their child after the program. Change in disease severity was not evaluated, as the authors stated the primary goal of the program to be reduction of anxiety about the disease.

### 3.2. Group Programs for Adult AD

Several studies have also evaluated multidisciplinary group interventions for adult patients with AD. Bostoen and colleagues evaluated a 12-week group-based education program for adults with AD and psoriasis [31]. The 12-week program consisted of 2-h sessions twice a week, including education on the skin disease, education on a healthy lifestyle, stress management techniques (exercise, yoga, mindfulness meditation), and medical feedback. Sessions were led by a dermatologist, dermatological nurse, pharmacist, dietician, training expert, psychiatrist, and psychologist. Fifty patients participated in the trial (21 with AD and 29 with psoriasis) and were randomized to the treatment group (which continued to receive medical therapy) or a control group receiving medical therapy only. Outcome variables including disease severity, quality of life, depressive symptoms, medical therapy use (*i.e.*, topical and systemic therapies), medical care costs (doctor visits and medication use), and cost-effectiveness of the program were collected at baseline and at 3-, 6-, and 9-month follow-ups. Analyses for patients with AD did not show any differences from the control group at follow-up, although the number of AD patients was small. Some improvements were seen for patients with psoriasis.

Ehlers and colleagues conducted a randomized controlled trial comparing 4 different group treatments for adults with atopic dermatitis to standard medical care [32]. The group treatments included: dermatological education (e.g., provision of information about the disease, the itch-scratch cycle, medications, allergies, and diet/nutrition, along with group discussion about barriers to treatment and individualized skincare instructions); relaxation training alone; cognitive-behavioral treatment (e.g., relaxation training, identification of itch-scratch triggers, distraction, habit reversal to substitute competing responses when the urge to scratch arises, modification of itch-related beliefs, and problem-solving); and multidisciplinary combined dermatological education and cognitive-behavioral treatment. Each group treatment consisted of 12 weekly sessions of 1.5 to 2 h each. At a 1-year follow-up, participants in the multidisciplinary group demonstrated greater improvements in skin severity than dermatological education alone or standard medical care, greater reductions in frequency of itching, frequency of scratching, and amount of topical steroid use than dermatological education alone, and greater reductions in catastrophizing/helplessness about itch than standard medical care. Of note, participants in the psychological treatment groups (relaxation, cognitive-behavioral treatment) achieved similar improvements in skin severity, frequency of itching and scratching, and catastrophizing about itch.

Evers and colleagues evaluated a brief group program led by a clinical psychologist/cognitive behavior therapist and a dermatologist nurse specialist, focusing specifically on coping with itch (e.g., self-monitoring, coping strategies to deal with itch/scratch triggers, and habit reversal to substitute competing responses when the urge to scratch arises) [33]. Sixty one participants attended four sessions conducted every two weeks, followed by booster sessions at 1 month and 3 months after the end of treatment. Changes in outcome measures were compared to changes for 30 patients on a waiting list for a similar duration of time, although patients were not randomized into these groups. At the end of the treatment period, relative to the control condition, group participants demonstrated greater improvement on itch intensity and duration (primary outcome), as well as skin severity, scratching behavior, itch-coping patterns (self-efficacy, catastrophizing) and illness cognitions. There were no significant differences between the groups on health-related quality of life. At 3- and 12-month follow-ups, the treatment group demonstrated sustained improvements in outcome measures and demonstrated reductions in dermatological healthcare use (e.g., outpatient and inpatient dermatology care and prescriptions for topical steroids and itch-relieving medications).

### 3.3. Multidisciplinary Care within a Day Treatment Program for Pediatric AD

Multidisciplinary care may also be provided through clinical programs for the treatment of patients with AD. Boguniewicz and colleagues describe a multidisciplinary day treatment program for children with AD who have failed outpatient care [34]. The program provides comprehensive evaluation, education and treatment over the course of 5–10 days. The treatment team consists of pediatric allergist-immunologists, a nurse practitioner, nurse educators, child life specialists, a creative art therapist, social workers, psychologists, a child and adolescent psychiatrist, and dieticians who collaborate to develop individualized plans for each patient. Education focuses on the chronic/relapsing nature of AD, exacerbating factors, therapeutic options, and details of the treatment regimen. Multiple educational strategies are used, including communication, direct demonstration, classroom teaching, and written materials. The authors note that direct demonstration of skincare by nurses and subsequent observation of patient/family technique is often useful for identifying errors in medication application and wet wrap therapy technique, as supported by a separate study by this group of authors, which found improvements in AD severity associated with education about and demonstration of wet wrap techniques [35]. Patients also receive education regarding potential skin irritants and are evaluated for potential environmental and food allergens, with a dietician available to provide education regarding appropriate diets. A psychologist or social worker evaluates child and family coping with illness and provides support as needed. Psychosocial treatment also includes support for managing scratching with techniques such as distraction, replacement, cognitive therapy, biofeedback, and hypnosis, depending on the developmental level and needs of the child. A psychiatrist is available for consultation regarding sleep problems and treatment options for concerns such as anxiety, irritability, and depression. Children and parents attend daily group therapy. The creative art therapist works with children on acclimating to treatment and understanding feelings about the condition, while parents may discuss parenting challenges and family coping with illness. At discharge, team members meet with the family to present and discuss a written home care plan [34,36].

### 3.4. Multidisciplinary Care within an Outpatient Program for Pediatric AD

Our program, the Atopic Dermatitis Center (ADC) at Boston Children’s Hospital, is an outpatient specialty clinic that provides multidisciplinary care for patients with severe AD who have not responded to conventional treatment alone. The mission of the ADC is to enhance care and quality of life for patients and their families through a collaborative approach to disease management. The ADC team is made up of a pediatric allergist, pediatric nurse practitioner, psychologist, and nutritionist. Patients are identified primarily through referrals from pediatricians, allergists and dermatologists. Patient history is obtained via medical records and parent-report measures assessing AD severity and AD-specific quality of life. Patients are screened by the nurse practitioner based on disease course and impact on quality of life.

Providers collaborate to provide each family with an individualized, coordinated treatment plan. The nurse practitioner and allergist evaluate for potential triggers such as food and environmental allergens, develop a comprehensive AD action plan [37], and address concerns about side effects. The nurse practitioner provides extensive education about AD, including the chronic nature of the disease, exacerbating factors, and the rationale for various elements of the management plan in improving the skin barrier function. Commonly, the nurse practitioner demonstrates skin care techniques, such as application of moisturizer and wraps. The psychologist develops strategies to enhance the child’s developmentally appropriate involvement in skincare, address barriers to adherence, break the itch-scratch cycle (e.g., behavioral alternatives to scratching, distraction, relaxation/stress management), manage sleep disruption (e.g., sleep hygiene, behavior plans) and address the impact of AD on mood and self-esteem. The nutritionist obtains a detailed diet history, provides recommendations to optimize growth and nutrition, reviews elements of food allergy management, and provides individualized recommendations for allergen-free foods, recipes, and substitutions. At the end of each visit, families are provided with a written AD action plan detailing how to respond to changes in disease status and when to seek additional medical help. Generally, children with severe disease are followed monthly, children with mild to moderate AD every 3 months, and children with well-controlled AD semi-annually or annually.

We have previously reported on chart review data from a sample of 69 patients followed in the ADC during an 18-month period soon after our program was established, focusing on parent acceptance of the integration of psychological support into the medical care of children with AD [38]. Children ranged in age from 2 months to 15 years, with the majority pre-school age or younger. On a physician/nurse practitioner rating of AD severity, over half of the children were assessed to have severe AD, over a third moderate AD, and a minority mild AD. Prior to the initial visit, parents rated the frequency of common AD-related behavioral concerns (itching/scratching, difficulty sleeping, skin picking, poor child cooperation with treatment, worry about side effects, difficulty following treatment recommendations, feeling overwhelmed by the condition) on a questionnaire developed for clinical use in our program. Parents’ initial request for a meeting with the program psychologist was not related to child disease severity, but was associated with child sleep problems and the parent feeling overwhelmed by the condition and having difficulty following treatment recommendations [38].

We have also utilized chart review data to examine factors associated with improvement in disease severity in our clinic population [39]. Over a 5-year period, data was collected from 170 patients ranging from 4 months to 16 years. At each clinic visit, AD severity was scored by the allergist or nurse practitioner. Parents rated the frequency of behavioral concerns in children with AD and their families prior to initiation of treatment and at follow-up clinic visits. Over 80% of patients demonstrated improvements in severity scores over time, with the full sample demonstrating a median 71% improvement in severity score averaged over clinic visits. The greatest improvement in disease severity occurred after the first visit to the clinic. Predictors of positive clinical outcome included more severe AD at initiation of treatment, younger child age at initiation of treatment, and improvement in parent report of difficulty following the treatment routine over the course of treatment. Reductions in severity correlated with decreased itching and improved patient sleep [39].

More recently, our group used chart review methodology to examine changes in disease severity from the initial multidisciplinary clinic visit to the first follow-up visit for new patients seen over the course of a 2-year period, using clinician ratings of skin severity on the Eczema Area and Severity Index (EASI) [40] and parent report on the Patient Oriented Eczema Measure (POEM) [41], which includes items about both the physical condition of the skin and associated life impairment (e.g., sleep, itch). This chart review was approved by the Institutional Review Board at Boston Children’s Hospital. Out of 69 patients seen for an initial visit within the 2-year period, 40 patients had one or more follow-up visits (with the remainder either returning to their original provider after a consultation visit, not following up as recommended, or not yet reaching their follow-up appointment date). Patients ranged in age from 6 months to 18 years. There were no differences in baseline POEM or EASI scores for patients who did or did not return for a follow-up visit. For patients with available EASI and POEM data at baseline and the first follow-up visit (*n* = 34), the mean duration of time between the baseline and follow-up visit was 2.8 (SD = 2.0) months. POEM scores decreased significantly from baseline to follow-up (*p* < 0.001) and EASI scores demonstrated a trend towards improvement (*p* = 0.061).

Table 1 provides a summary of study methodology, intervention type, and reported outcomes for studies of multidisciplinary interventions for atopic dermatitis.

**Table 1:** Summary of outcomes for studies of multidisciplinary interventions for atopic dermatitis.

Authors	Study Design/Intervention	AD Severity	Itching and Scratching	Itch-Coping Patterns	Quality of Life (Qol)	Healthcare Use/Cost	Adherence
Staab *et al*. [27]; 2002	Randomized controlled trial	No significant difference in change in SCORAD ratings between treatment and control groups	Not assessed	Not assessed	Greater improvement in confidence in treatment for treatment group; no other significant differences in QoL between treatment and control groups	Greater reduction in medical consultation and prescription costs for treatment group	More consistent skincare and greater improvement in skincare adaptation based on skin severity (appropriate use of steroids, antiseptics) for treatment group
	Group educational program for parents *vs.* waitlist control condition						
Staab *et al*. [29]; 2006	Randomized controlled trial	Greater improvement in SCORAD ratings for treatment group	Not assessed	Greater reductions in catastrophizing (8–12 and 13–18 year olds) and improvement in coping (8–12 year olds) for treatment group	Greater improvement in QoL for treatment group	Not assessed	Not assessed
	Group educational program for parents and pediatric patients *vs.* waitlist control condition						
Ricci *et al*. [30]; 2009	Pre-post comparison	Not assessed	Not assessed	Not assessed	Improvement in parental and child QoL from start to end of treatment	Not assessed	Not assessed
	Group educational program for parents						
Bostoen *et al*. [31]; 2012	Randomized controlled trial	For AD patients, no significant difference in change on EASI or SCORAD ratings between treatment and control group	Not assessed	Not assessed	For AD patients, no significant difference in change in QoL scores between treatment and control group	No significant differences in medication use or health-care costs (medications, doctor visits) between treatment and control groups	Not assessed
	Group education program for adult patients with AD or psoriasis *vs.* usual medical therapy						
Ehlers *et al*. [32]; 1995	Randomized controlled trial	Greater improvement in severity (body surface affected and lesion severity) for DEBT group than DE or standard medical care	Greater reduction in itching and scratching frequency for DEBT than DE; no significant differences in itch/scratch intensity between groups	Greater reduction in catastrophizing than standard medical care	Not assessed	Greater reduction in topical steroid use for DEBT group than DE alone	Not assessed
	4 different group treatment programs * for adult patients *vs.* each other and standard medical care						
Evers *et al.* [33]; 2009	Controlled trial	Greater improvement in EASI ratings for treatment group	Greater reductions in itch intensity and duration and scratching frequency and duration for treatment group	Greater reductions in catastrophizing and improvement in self-efficacy for treatment group	No significant differences in change in QoL between treatment and control groups	Reductions in dermatology visits and medication use for treatment group	Not assessed
	Group itch-training intervention for adult patients *vs.* waitlist control condition						
Boguniewicz *et al*. [34]; 2008	Descriptive	Not assessed	Not assessed	Not assessed	Not assessed	Not assessed	Not assessed
	Day treatment program for pediatric patients						
LeBovidge *et al*. [38]; 2006	Descriptive	Not assessed	Not assessed	Not assessed	Not assessed	Not assessed	Not assessed
	Outpatient program for pediatric patients						
Chou *et al*. [39]; 2011	Descriptive/chart review	Majority of patients demonstrated improvement in EASI ratings from baseline to follow-up visits	Not assessed	Not assessed	Not assessed	Not assessed	Not assessed
	Outpatient program for pediatric patients						

Abbreviations used: EASI (Eczema Area and Severity Index) [40], SCORAD (Scoring Atopic Dermatitis) [42]; * Four groups include: DE = dermatological education program, AT = autogenic training (relaxation therapy alone); BT = cognitive-behavioral therapy, DEBT = multidisciplinary combined DE and BT.

## 4. Discussion

Multidisciplinary interventions for patients with AD typically include several common elements, including intensive education about the condition and its management, psychological and behavioral interventions, and particularly in the case of pediatric patients, nutritional support. The aims of such programs are to provide families with the tools and knowledge needed to effectively manage AD and improve quality of life. Among studies included in this review, most demonstrated improvements in skin severity associated with participation in multidisciplinary treatment [29,32,33,39], although this was not universal [27,31], and some studies did not assess this outcome [30,34].

A major goal of multidisciplinary care is to improve adherence to time-intensive medical routines. Across interventions included in this review, education about AD typically involved teaching about the course and triggers of the disease, modeling treatment techniques, eliciting and discussing parent concerns about treatment side effects, and providing written AD action plans. Additionally, psychologists were involved in problem-solving barriers to adherence and facilitating children’s age-appropriate involvement in skincare. Only one study in the review, however, directly assessed changes in treatment behavior associated with multidisciplinary interventions for AD. Staab and colleagues found improvements in consistency of skincare and appropriate adaptation of treatment to the severity of the skin for parents participating in a multidisciplinary group training program [27]. Parents in the intervention group also reported greater satisfaction with medical treatment associated with participation in the intervention. Data from our outpatient program for children with AD found that reductions in parents’ perceived difficulty with following treatment recommendations was correlated with improved clinical outcomes, underscoring the importance of identifying and addressing aspects of the treatment regimen that are challenging for each family [39]. Such education practices are consistent with recent recommendations for use of therapeutic patient education to improve adherence in patients with AD by focusing on improving families’ knowledge about the condition, addressing barriers to adherence, and developing skills to self-assess disease status and adapt treatments as necessary [43]. Indeed, a recent review suggests that multidisciplinary interventions are currently being utilized to provide therapeutic patient education for patients with AD in a number of hospital-based centers worldwide, although the majority of programs have not published data on clinical outcomes, and thus were not included in this review [44].

Findings regarding the impact of multidisciplinary interventions on quality of life were mixed. This may result in part from differences in methodology for assessing quality of life across studies. Of note, each of the three studies assessing interventions for pediatric AD reported positive changes in quality of life [27,29,30] from the parent perspective, reflecting positive impact of multidisciplinary interventions on family quality of life. This is an important outcome, given significant family stress associated with management of AD [10].

Multidisciplinary programs included in this review showed strong promise in helping patients and families cope with itch, which has a significant impact on quality of life. In addition to managing itch through medications and disease control, patients and families participating in multidisciplinary interventions for AD are taught specific cognitive-behavioral strategies, such as identification of itch-scratch triggers, development of behavioral alternatives to scratching, use of relaxation techniques to modify perception of itch and address the itch-stress link, and modification of itch-related beliefs. Children and adults participating in group educational programs have demonstrated reduced intensity and frequency of itching and scratching, reduced catastrophizing about itch, and increased confidence in the ability to cope with itch [29,32,33].

Psychological components of multidisciplinary interventions for AD typically also include education about strategies to address sleep disruption due to itch and learned behavioral patterns, although this was not directly assessed in most studies. In our outpatient program, we found improvement in parent-report of both itch and sleep, associated with improvement in skin severity from the initial clinic visit to the first follow-up [39]. These outcomes were likely achieved through both improved disease control and use of behavioral strategies. Importantly, our experience has been that integration of psychological care as a “standard” component of multidisciplinary treatment of AD increases access to psychological support and may reduce stigma associated with mental health care.

While findings suggest that multidisciplinary interventions for AD do achieve important aims, such as improving disease control and helping to break the itch/scratch cycle, there are limitations to existing studies and important avenues for further research. Studies included in this review did not all assess the same outcomes, making it difficult to directly compare results across interventions. The multidisciplinary clinical programs described in the literature (outpatient clinic, day treatment program) have not yet been studied using randomized controlled trials, so it is not possible to compare outcomes to standard medical treatment of AD [34,39]. However, these programs specifically treat patients who have not responded to conventional medical treatment of AD and are experiencing a significant impact of the disease on quality of life, suggesting benefits of the multidisciplinary model for patients with treatment-resistant disease.

Management of AD can pose a significant financial burden for families [6,45]. Several studies included in this review suggested reduced economic burden and/or dermatological healthcare use for patients and families participating in multidisciplinary AD interventions, compared with standard medical treatment [27,32,33]. However, results were not consistent, with one study finding no differences in medication use or healthcare costs between the treatment and control groups [31]. More research is needed to better understand the cost-benefit analysis of providing multidisciplinary care for AD, given both high costs of treatment for severe AD, as well as the time and resource intensive nature of multidisciplinary treatments [44]. It may also be useful to evaluate which elements of multidisciplinary treatments are most critical for improved outcomes, in order to streamline interventions when resources are limited. Research in our multidisciplinary outpatient clinic indicated that the greatest improvement in disease severity occurred following the first visit, when families received a comprehensive written AD action plan, a nutritional plan for selected allergen avoidance, and behavioral coping strategies, suggesting that when resources are limited, even shorter-term multidisciplinary intervention may yield significant benefits [39]. In future research, it may also be helpful to compare results of multidisciplinary programs including interventions for children with those aimed at parents alone. Our clinical experience suggests the importance of empowering children to take an active, developmentally appropriate role in performing AD skincare and coping with itch.

It is also important to consider which patients may benefit most from multidisciplinary approaches. Studies of group educational training programs included in this review generally enrolled patients with moderate to severe AD [27,29,30], and multidisciplinary clinical programs reported strong candidates to include those who had failed conventional medical treatments, had experienced recurrent skin infections, were concerned with treatment side effects, reported a significant impact of the disease on quality of life, and/or were avoiding multiple foods due to allergy concerns [34,39]. Chart review data from our multidisciplinary outpatient program indicated that parent reports of sleep disruption, feeling overwhelmed by managing the condition or having difficulty following treatment recommendations were predictive of family interest in the multidisciplinary model, while disease severity was not [38]. Similarly, a study conducted by Schut and colleagues found that parental dissatisfaction with the child’s standard medical care for AD, active problem-solving, and low social support predicted interest in patient education programs for AD, while disease severity did not [46].These findings confirm the importance of assessing the impact of AD on quality of life, rather than basing decisions about the need for multidisciplinary care on AD severity alone. Findings from our outpatient program also suggest that families benefitted most when treatment was initiated early in the disease course. This may reflect the natural history of the disease; however, it has been our impression that introducing behavioral changes and medical interventions earlier can help to control the disease and break the itch-scratch cycle [39].

## 5. Conclusions

In summary, despite limitations to the current research, available evidence supports the use of multidisciplinary intervention approaches for patients with AD and their families, as a model of treatment or an adjunct to standard medical care. Further research will be useful in assessing the impact of multidisciplinary interventions on important outcomes such as treatment adherence and sleep, identifying the elements of multidisciplinary interventions that are most critical for improved outcomes, and identifying the best candidates for multidisciplinary intervention approaches.
